# Case Report: An Atypical Presentation of Panic Disorder Masquerading as Possession Trance

**DOI:** 10.3389/fpsyt.2021.819375

**Published:** 2022-01-12

**Authors:** Howard C. H. Khoe, Alakananda Gudi

**Affiliations:** Department of Psychiatry, Singapore General Hospital, Singapore, Singapore

**Keywords:** Chinese, culture-bound syndrome, panic disorder, possession trance, psychiatry, religion

## Abstract

This case report demonstrates an atypical presentation of panic disorder which masqueraded as episodes of possession trance. Patient X is a 62-year-old Chinese female who presented with recurrent episodes of uncontrollable screaming and shaking of all four limbs. During these episodes, she reported auditory hallucinations (Buddhist chanting) and visual hallucinations (a rotting corpse) which she attributed to the influence of “evil spirits.” She was diagnosed with panic disorder with culture-specific symptoms on a background of major depressive disorder. With an understanding of the patient's belief system and an empathetic approach during psychoeducation, she eventually accepted the use of pharmacotherapy. She was prescribed escitalopram (started at 2.5 mg OM) and clobazam (10 mg ON) with good clinical effect and cessation of episodes afterward.

## Introduction

Possession trance is a common culture-bound syndrome in Singapore. Owing to phenomenological similarities, possession trance disorders are classified under dissociative identity disorder (DID) in DSM V(1–4). It describes a state with an alteration in the level of consciousness, amnesia during the trance, a stereotyped behavior characteristic of a deity, duration of less than an hour, normal behavior in between trances, and fatigue upon termination (5). Due to their similar presentations, differentiation between possession trances and panic disorders with culture-specific symptoms can be diagnostically challenging. This case report demonstrates an atypical presentation of panic disorder which had masqueraded as episodes of possession trance, highlighting the subtle differences between the two.

## Case Presentation

A 62-year-old Chinese female, referred here as patient X, was admitted for recurrent episodes of uncontrollable screaming and shaking of all four limbs.

These episodes began 4 days before admission, occurring two to three times daily. Each episode was abrupt in onset, lasting several minutes to an hour. Onset was characterized by an uncomfortable coldness in the chest spreading outwards to both arms and legs, transforming into a tingling sensation followed by involuntary tremors. Patient X described a lack of control, oscillating between laughing and crying whilst shouting incomprehensibly and flailing her limbs against her surroundings. She experienced fatigue and diaphoresis with no loss of consciousness nor amnesia during these episodes. She had persistent concerns over future episodes, which she described as unbearable.

In the emergency department, patient X suffered an episode and was given intramuscular haloperidol 5 mg with cessation of the episode within minutes. She was able to sit up and apologize, before cooperating with further investigations. After 10 min, she suffered another episode and was given intravenous midazolam 1 mg with the resolution of symptoms within minutes.

Similar episodes had occurred 3 years ago after having undergone a left open radical nephrectomy for xanthogranulomatous pyelonephritis. Morphine was administered intra-operatively and tramadol post-operatively for pain control and the neurologist's impression was “episodic hyperkinetic movement disorder possibly secondary to opioids.” Investigations including electroencephalography (EEG) and MRI brain imaging were normal. Tramadol was discontinued and she was started on oral clobazam 10 mg TDS. These episodes ceased after 2 weeks and she was discharged with a tailing regimen of Clobazam. During the same post-operative period, she reported low mood and fleeting suicidal thoughts resulting from her various medical conditions. She was diagnosed with Major Depressive Disorder (MDD) and started on escitalopram 5 mg ON which was titrated upwards to 10 mg ON during her follow-up appointments. She remained stable on this dose for 2 years before discontinuing her medications.

Patient X had no significant personal or family history of neurological or psychiatric illnesses. Her other medical conditions include essential hypertension, hyperlipidemia, and diabetes mellitus. She had a good relationship with her husband and two children. Her pre-morbid personality was described by her husband as “optimistic and sensitive” and during interviews, she was found to be agreeable with no evidence of having histrionic or borderline personality disorder traits. She underwent formal schooling till the age of thirteen and worked blue-collar jobs before becoming a housewife. She denied smoking, alcohol consumption, and other illicit substances misuse.

On admission, vital signs were normal with no neurological deficit on physical examination. Patient X was neatly dressed with good eye contact and a pleasant disposition. She was euthymic with a reactive affect. Her speech was relevant, coherent, and appropriately paced. There was no evidence of formal thought disorders, hallucinations, or delusions. Investigations were unremarkable: computed tomography scan of her brain, EEG, and blood tests (thyroid function test, electrolytes, renal panel, liver panel, full blood count, folate, and vitamin B12 serum levels).

During her inpatient stay, patient X verbalized new passive suicidal ideations secondary to the distressing nature of these episodes. She denied features of depression and anxiety before admission and a corroborative history from her husband did not reveal any significant psychosocial stressors or depressive/anxious features. With rapport building and exploration of her personal spiritual beliefs, she revealed concerns of “evil spirits.” She opined that her radical nephrectomy operation in 2017 was delayed because “the evil spirits were after me.” She had previously heard the chanting of Buddhist mantras for an entire week following her operation and had also seen a “rotting corpse” on her bed. She believed supernatural forces had caused these episodes, which resolved after taking medications and having received blessings from her religious leaders. During subsequent reviews, she revealed these episodes first began 30 years ago after an abortion and had lasted for a week. While her husband supported her decision, she nonetheless felt guilt and shame, citing financial constraints as the reason for the abortion.

Patient X was diagnosed with panic disorder with culture-specific symptoms on a background of MDD. She was restarted on escitalopram 2.5 mg OM (with plans to increase the dose further at follow-up clinics) and clobazam 10 mg ON and responded well-before being discharged.

## Discussion

The distinction between possession trance and panic disorder is crucial as management is vastly different.

A Singapore study (5) on trance states showed that trance possessions were precipitated by anger and frustration, which are also known triggers for panic attacks. This is based on the theory that trances embody and convey distress arising from feelings of anger, grief, fear, and vulnerability precipitating from stressful experiences. The treatment of trance possessions targets such issues through counseling and psychotherapy (6–9) exploring underlying stresses and alternative coping strategies. On the contrary, panic disorders are treated with pharmacotherapy and/or psychotherapy. First-line treatment includes selective serotonin reuptake inhibitors (antidepressants) as recommended by the NICE 2011 guidelines (10) and a short course of benzodiazepines by the American Psychiatric Association guidelines (11).

In Singapore, 22% of Chinese psychiatric patients (12) felt that they were possessed by spirits that had caused them to behave and think abnormally and amongst patients with trance (5) 40% experienced auditory hallucinations and 32.7% had visions of spirits and shadows. The religious and cultural overlay to psychiatric presentations is not unique to Singapore and has been observed in other parts of the world: Bourguignon (13) analyzed samples from 488 societies and found that 90% of societies displayed trance and/or possession. Bragazzi NL (7) also described a clinical case of a Muslim girl reporting possessions and panic attacks in Italy. Djinns are “evil creatures” as described in the Qur'an and in the case study, the 19-year-old Muslim Italo-Tunisian girl reported panic attacks with djinns invading her body and mind soon after she emigrated to a different country. The patient was successfully treated with fluoxetine 20 mg/daily and psychological counseling and psychotherapy.

At first glance, it may seem that patient X's professed religious beliefs and episodes of uncontrollable screaming, shaking of all four limbs, and hallucination are typical of a trance possession, however, a closer examination of her symptoms reveals otherwise.

Each episode was characterized by an abrupt surge of intense fear that reached a peak within minutes and was accompanied by 6 of 13 DSM V criteria (2) for panic disorder: sweating, trembling and shaking, chest discomfort, chills sensations, paraesthesia in her limbs and fear of losing control. These episodes were recurrent and unexpected with patient X showing a persistent concern of additional attacks. These episodes were not attributable to the physiological effects of a substance or another medical or mental condition. While there were no clear traumatic experiences behind her current episodes, her previous abortion and nephrectomy may be potential triggers of deep psychological disturbances that may act as a precipitating factor for her panic attacks. Hecker (14, 15) found that pathological spirit possession might be seen as a trauma-related disorder, culturally determined through the interpretation of dissociative symptoms related to traumatic exposure. In a similar parallel, patient X's reporting of supernatural possessions as explanations for her episodes could be a culturally determined phenomenon through her interpretation of her panic disorder symptoms. In the context of her Buddhist beliefs, the hallucinations of a rotting corpse may be interpreted as a symbol of the guilt and shame she experiences over her previous abortions and chanting of Buddhist mantras a corresponding prayer for help. Attending to the patient's phenomenological experiences in future sessions would aid in managing her panic disorder.

In addition, there was no disruption in identity, alteration of consciousness, or gaps in the recall of events, making a diagnosis of possession trance even less likely. The fact that she had a recollection of her idiosyncratic behaviors is in stark contrast with most possession trance episodes, which is usually accompanied by amnesia. Therefore, the uncontrollable screaming, hyperkinetic movements, auditory and visual hallucinations on a background of espoused religious beliefs was a red herring. In the context of her other symptoms, these should instead be interpreted as culture-specific symptoms of a panic attack. Depression with psychotic features is less likely given the absence of depressive symptoms, with the onset of passive suicidal ideations only after panic attacks began. Her Montgomery-Asberg Depression Rating Scale score of 8 (depressive symptoms absent) also goes against a diagnosis of MDD. Furthermore, the transient nature of each episode (less than an hour) with an immediate return to baseline mental status and retained insight is uncharacteristic of a brief psychotic episode. Her normal EEG findings (during both current and previous admissions) and normal brain imaging findings, make a diagnosis of temporal lobe epilepsy or other intracranial organic pathologies less likely.

Limitations of this study include a lack of toxicology screen performed (patient and corroborative history from husband suggested no natural herbs/substance misuse and hence it was not performed) and its nature as a case report.

## Concluding Remarks

While there has been one other case study describing possessions and panic attacks in a Muslim girl, this is the first case study to our knowledge of a Chinese patient with Buddhist beliefs presenting with an atypical presentation of panic disorder with culture-specific symptoms masquerading as possession trance.

There are race-ethnic differences in the rates of panic disorder, panic attacks, and certain panic attack symptoms (16) (e.g., White Americans have a higher frequency of heart-racing than Asian Americans) and possible reasons for cross-cultural differences lies in the different meaning and emotional salience of panic attack symptoms within the context of each culture (17). Therefore, an empathetic and clear understanding of the patient's cultural beliefs would allow the attending psychiatrist to better appreciate the varied presentations of common psychiatric conditions.

In Asian societies, psychiatric patients commonly consult traditional healers rather than western-trained doctors. Seeking spiritual help may help rather than hinder a patient's mental health and various studies have supported this claim (18). For example, patients with schizophrenia who spent more time in religious activities tended to have a better prognosis at a 2- and 5-year mark in follow-up studies (19, 20). van Duijl et al. (21) found that explanatory models based on spirit possession with traditional healing processes of spirit possessions eventually led to significant improvement for 99% of patients, highlighting the important complementary role traditional healers can play in the provision of local mental health care services. In this case, we acknowledged her plans to seek a religious consult and worked alongside her belief system while counseling on the benefits of complementing her treatment with prescribed pharmacotherapy to aid with the anxiety surrounding each attack. By demonstrating an acceptance of the patient's interpretation of her symptoms, rapport was further built and she was agreeable to re-initiate pharmacotherapy immediately and reconsider psychotherapy at subsequent follow-up appointments.

In summary, this case report highlights the importance of recognizing the patient's cultural beliefs and how they may lead to culture-specific symptoms with unique presentations of common psychiatric conditions. Demonstrating an understanding of the patient's belief system can lead to a therapeutic alliance and increase a patient's compliance with the prescribed management plans.

## Data Availability Statement

The original contributions presented in the study are included in the article/supplementary material, further inquiries can be directed to the corresponding author/s.

## Ethics Statement

Written informed consent was obtained from the patient for their anonymized information to be published in this article.

## Author Contributions

HK obtained consent and wrote the manuscript. AG identified the case and supervised the writing of the manuscript. Both authors contributed to the article and approved the submitted version.

## Conflict of Interest

The authors declare that the research was conducted in the absence of any commercial or financial relationships that could be construed as a potential conflict of interest.

## Publisher's Note

All claims expressed in this article are solely those of the authors and do not necessarily represent those of their affiliated organizations, or those of the publisher, the editors and the reviewers. Any product that may be evaluated in this article, or claim that may be made by its manufacturer, is not guaranteed or endorsed by the publisher.
